# I can look for it! Modulation of a concurrent Visual Working Memory task in Visual Search in development

**DOI:** 10.3389/fpsyg.2022.907121

**Published:** 2022-07-22

**Authors:** María Quirós-Godoy, Beatriz Gil-Gómez de Liaño, Elena Perez-Hernandez

**Affiliations:** ^1^Department of Social Psychology and Methodology, Universidad Autónoma de Madrid, Madrid, Spain; ^2^Department of Development and Educational Psychology, Universidad Autónoma de Madrid, Madrid, Spain

**Keywords:** visual search, working memory, strategies, children, development, attention, executive functions, Theory of Constructive Operators

## Abstract

Daily classroom activities that require children to perform visual search (VS) tasks are common across all educational levels: from searching for a missing piece of a puzzle in kindergarten to solving equations in college. However, VS tasks are often not performed in isolation, but rather students are maintaining information related to an ongoing task that loads working memory (WM). Unfortunately, it is still unclear how these processes interact and evolve in development. The present work aims to study how a concurrent visual WM (VWM) load can modulate VS performance based on the Developmental Model of Endogenous Mental Attention (Pascual-Leone and Johnson, 1999, 2005, 2021). A sample of kindergarten, elementary (2nd and 4th grades), middle school (6th grade), and college students looked for real-world photorealistic targets while maintaining similar objects in VWM in a dual-task paradigm. VWM load was manipulated using high and low memory load conditions. Additionally, looking for potential modulations related to individual differences, we studied the relationship between IQ, VWM span, and executive functions with VS efficiency. Finally, we also registered reported measures of potential strategies employed during the VS task. The results from a large sample of 147 participants between 5 and 25 years old revealed that even the youngest children could efficiently perform a VS task with a concurrent VWM load, replicating previous results found in adulthood. However, we found a slight increase in false alarms and commission errors when memory was highly loaded for all the participants regardless of age. As expected, we found positive correlations between VS efficiency and IQ and VWM span measures. Interestingly, the proportion of participants who used tracking organization strategies increased with age in all cases. However, although cognitive strategies to remember the target became more complex as age increased, it was only significant under the low VWM load conditions. The results seem relevant to understanding the development of VS based on the Model of Endogenous Mental Attention and the design of training programs to improve attention. The implications in educational contexts are discussed and are especially relevant for students with learning disabilities or attention problems.

## Introduction

Visual search is the basis of many activities daily performed in educational contexts. In kindergarten, children look for the necessary pieces to finish a tower of blocks or the red color among several paintings to draw the path in a maze. In primary school, children must look for spelling errors in grammar writing exercises or search for the isosceles triangle among other types of triangles in math exercises. Visual search is even present at advanced levels of education, i.e., when solving equations in college. These visual search tasks are often not performed in isolation, but rather students are maintaining information related to an ongoing task that can load working memory. These evolving processes improve as time passes and the cognitive system matures. Because of the importance of these two processes at all levels of education in school contexts, we will study how a concurrent working memory load task can modulate visual search efficiency in development within a large sample of 147 observers from 5 to 25 years old and structured on a neurodevelopmental perspective under the Model of Endogenous Mental Attention (Pascual-Leone and Johnson, 1999, 2005, 2021).

Visual search has usually been studied in cognitive development using two essential paradigms: feature and conjunction search tasks. In feature search (e.g., looking for a red pencil among blue pencils), the target is easily detected as it is defined by one feature that differentiates it from the distractors (color in our example: red vs. blue). Basic automatic processes capturing attention seem to be the base of feature search, and they have been found present in early stages of development (Gerhardstein and Rovee-Collier, 2002). However, for conjunction search, the target shares one or more features with the distractors (e.g., finding a red pencil among red pens and blue pencils), making it more challenging to search and thus requiring a more controlled and guided attentional process. Studies have shown variable results in conjunction search tasks, from set size functions clearly decreasing with age until adulthood (Donnelly et al., 2007; Michael et al., 2013; Woods et al., 2013; Brennan et al., 2017) to smoother non-dramatic set size modulations in the lifespan (Hommel et al., 2004). According to results, most researchers have postulated executive functions, specifically attentional control, as the base to explain the results. Michael et al. (2013) and Woods et al. (2013) concluded that the executive functions needed to perform a visual search efficiently are in development during childhood, explaining the modulations. Namely, they must rely on inhibitory-attentional control, mental flexibility, planning, and working memory processes to perform a conjunction search-like, more complex search task. Indeed, a recent developmental study using conjunction search-like inefficient although more ecological, unique object search has shown visual search as a powerful paradigm to understand executive function development. Gil-Gómez de Liaño et al. (2020) found different developmental trajectories for accuracy, search slopes, and intercepts in visual search closely related to underlying executive functions described in Anderson’s (2002) neuropsychological executive function model. The developmental course of accuracy essentially overlapped attentional control in Anderson’s model, while slope functions overlapped goal setting, and intercepts followed the time course of cognitive flexibility and information processing.

Working memory seems to be a key executive function process in visual search. The attributes of the target that guide search, conceptualized as what Wolfe (2021) calls the “*guiding template*,” must be stored in working memory. Although with distinct hints other researchers have supported a similar viewpoint. Olivers et al. (2011) argue that the “*target template*” in visual search is probably maintained in working memory. Since working memory seems to play a crucial role in understanding guidance in visual search complex tasks like conjunction search or unique object searches, comprehending its development could be essential to understand differences in visual search among children of varying ages. How does performing a concurrent working memory task might affect visual search efficiency throughout development? Could working memory load differently affect performance in visual search in diverse developmental stages?

Working memory is known to be under development between 4 and 12 years old. Its development is closely related to the maturation of the prefrontal cortex, with significant changes between 6 and 10 years old and the age of 9–10 being a hallmark in working memory development (Luciana and Nelson, 1998; Brooking et al., 2012; Simmering, 2012; Vuontela et al., 2013). However, most of these studies are based on models conceptualized from research on adults’ working memory (e.g., models from Baddeley and Hitch, 1974; Cowan, 1988; Baddeley, 2000). These models have a limitation of not offering a developmental perspective. As Karmiloff-Smith (1995) proposed, we should focus on the “*changes over time*” of a developing mind rather than only when the child reaches adult levels in a given cognitive process.

An influential model studying attention in children that follows the developmental viewpoint suggested by Karmiloff-Smith is the Model of Endogenous Mental Attention proposed in the Theory of Constructive Operators (Pascual-Leone, 1970, 1995; Pascual-Leone and Johnson, 2005, 2021). Interestingly, our study pays special attention to understanding the development of controlled and effortful attention, executive attention, from a neurodevelopmental perspective.

According to the Theory of Constructive Operators, schemes are the basic unit of information, and they are expressed as neural networks in the brain (Pascual-Leone and Johnson, 2005; Arsalidou et al., 2019). Schemes can be of different types (figurative, operative, or executive), and all of them are under the regulation of domain-free operators, “functional mechanisms of brain hardware” (Pascual-Leone and Johnson, 2005, 2021). Each operator is related to a particular brain region, has a specific function (Arsalidou et al., 2019), and can be applied to schemes in any content (e.g., visual or auditory, Pascual-Leone and Johnson, 2005, 2021). Although Pascual-Leone and Johnson (2021) described eleven operators, we will only mention those applicable to the Model of Endogenous Mental Attention and directly related to the so-called Mental Attention potentially affecting the processes involved in a visual search task, that is, the M, I, E, and F operators (Pascual-Leone, 1995; Pascual-Leone and Johnson, 1999, 2005, 2021; Arsalidou et al., 2019).

**FIGURE 1:**
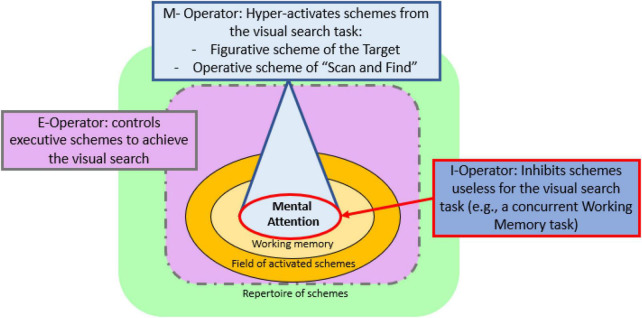
Diagram of the functioning of the mental attention during a visual search task, based on the visual representations of the Model of Endogenous Mental Attention from Arsalidou et al. (2019) and Pascual-Leone and Johnson (2021).

Considering a visual search, we show in Figure 1 a diagram of the Endogenous Mental Attention and operators that impact the efficiency of the task. The M-operator (mental attentional activation) is responsible for the effort of fully hyper-activating the necessary schemes to perform a task. In our visual search case, it is the figurative scheme of the “target” and the operative scheme of “scan and find.”

On the contrary, the I-operator (mental attentional inhibition) inhibits unwanted schemes that could lead to a task error. The Theory of Constructive Operators distinguishes between effortful inhibition and automatic inhibition (Howard et al., 2014; Pascual-Leone and Johnson, 2021). The automatic inhibition deactivates schemes effortlessly, especially in facilitating tasks. The effortful inhibition suppresses hyperactivated schemes that are incompatible with successful performance and that, otherwise, would remain active within the focus of the Mental Attention. Specifically, in the visual search task, the I-operator must inhibit the distractors (the more features shared with the target, the greater the effort) or the schemes related to the concurrent tasks (in the case of the present experiment, schemes related to the concurrent working memory task). Poor functioning of one of the two operators, the M or the I, would cause a decrease in accuracy in the visual search task, that is, in the number of hits. Specifically, omissions would be related to a deficit in the M-operator due to insufficient activation of the operating scheme of the target itself. On the contrary, false alarms or commissions (confounding a distractor with the target) would be due to a problem with the I-operator, deactivating schemes incompatible with the task goal.

The E-operator controls activated executive schemes useful to achieve the task directly related to plans, goals, or strategies to perform the visual search. Therefore, a change in response speed or search slopes could be an indicator of the functioning of the E-operator. Moreover, studies on task efficiency (when controlling speed/accuracy trade-offs) can serve as a global measure of the performance of the I-, M-, and E-operators as a unit.

In the model, the E-, M-, and I-operators are related to the prefrontal lobe’s function. The fourth operator that might apply in a visual search task, the F-operator, organizes the *field of mental attention*, unifying mental representations from a neo-Gestaltist point of view. Finally, the Schematic Overdetermination of Performance principle synthesizes all the most dominant schemes to produce a task-directed behavior (yes/no motor response). The coordinated functioning of the M-, I-, E-, and F-operators is essential, especially in complex situations (like conjunction searches or unique object search) when the cognitive demand increases because of salient or irrelevant information that keeps activated schemes potentially advocating an inadequate response in a task (refer to Pascual-Leone and Johnson, 2005, 2021, for a deeper explanation about the Model of Endogenous Mental Attention). As we can see, the Model of Endogenous Mental Attention allows for us to study the cognitive processes underlying the visual search and their development.

On the other hand, the interaction between working memory and visual search has been a key research question in psychological science. A typical paradigm followed in many studies testing adults in the field studies how performance in visual search can vary under different conditions of working memory load in dual-task paradigms. However, the results of these studies with adult population are variable. In a review, Soto et al. (2008) showed how the contents of a concurrent memory load task can attract attention in attentional tasks like visual search. Also, Lavie et al. (2004) and Lavie and de Fockert (2005) found that high loads in a concurrent working memory task could impair efficiency in visual search probably because of competition between control cognitive resources to avoid distractors in the attentional task and memory contents. However, other studies have found somehow the opposite result: better performance in the attentional task under high visual working memory loads. Smilek et al. (2006) studied how cognitive strategies could influence visual search performance during similar dual-task paradigms. They found better search performance by increasing the difficulty of the memory task when the search strategy was instructed to be a “passive” search letting the target “pop out.” This effect disappeared when the search strategy was to actively search for the target. Also, Gil-Gómez de Liaño et al. (2011) and Gil-Gómez de Liaño et al. (2014) found similar results using Rapid Serial Visual Presentation tasks as a way of search in time rather than in space. However, many other studies have just simply not found any modulation of working memory loads/contents in attentional tasks, particularly in visual search tasks. Downing and Dodds (2004) and Gil-Gómez de Liaño et al. (2011) tested the effects of working memory contents overlapping the distracters during visual search tasks but failed to find any modulation of memory contents or any difference between high and low loads in search performance. Both studies support theories in which working memory is fractionated, allowing for the maintenance of non-essential items in the visual search task. Vogel et al. (2001) and Woodman and Luck (2007) also found similar results and argued that knowing that the items stored in working memory could never be a target in the search task, participants can strategically inhibit them during the search. Moreover, Gil-Gómez de Liaño et al. (2016) showed again similar “lack of effects” results non-replicating previous studies in a set of experiments increasing power and pointing toward the necessity of replication studies to increase the reliability of studies in the field. That study led to a meta-analysis (Quirós-Godoy et al., 2017) concluding that working memory load did not interact with visual search (and other attentional tasks like the flanker task or Stroop-like tasks) and supporting again that lack of interaction.

However, to our knowledge, there are no similar dual-task articles studying working memory and visual search interactions in children populations. A recent correlational study, though, points toward a potential modulation. Guilbert et al. (2020) found that better working memory abilities measured by Backward Digit Span let children from 8 to 11 years be more organized during a visual search in the Bells cancelation task. However, they did not study how different memory loads might affect visual search in children and how that could vary in development at different ages.

The Model of Endogenous Mental Attention (Pascual-Leone and Johnson, 2005, 2021) offers a neurodevelopmental perspective that could allow for us to understand working memory and visual search interaction development in different age stages. Under this model, working memory encompasses all hyperactivated schemes (e.g., schemes boosted by other operators like motivational or affective schemes) including those boosted by Mental Attention in coordinated studies on the M- and I-operators (Pascual-Leone, 2000). That is why the development of the M-operator and the I-operator plays a relevant role in explaining working memory development. The capacity of the M-operator is limited. It has a maximum number of schemes that it can keep effortfully active at a time. Empirical data show that M-capacity increases by one scheme every 2 years from about 3 years old to adolescence (15–16 years). In this developmental stage, the M-operator reaches its maximum of seven simultaneous schemes at once. Added to this is “e,” which refers to the capacity developed during the sensorimotor stage before 3 years of age (Pascual-Leone and Johnson, 2005, 2021; Arsalidou et al., 2019).

On the other hand, the development of the I-inhibitor also distinguishes between the automatic inhibition and the effortful inhibition, as previously mentioned: while at 7-years of age one can automatically inhibit as effectively as adults, in the case of the effortful inhibition, there is a gradual improvement in adulthood until older ages (even 12-year-olds perform significantly worse than adults; Howard et al., 2014).

Finally, and although secondary to the main objectives of this study, it is important to mention that other cognitive processes might interact with visual search performance (Michael et al., 2013; Woods et al., 2013) that we should consider. In the Model of Endogenous Mental Attention framework, Arsalidou et al. (2019) described a relationship between Mental Attention and fluid intelligence. Navarro et al. (2006) and Howard et al. (2013) found that gifted children had higher M-measurements than their non-gifted peers. A higher M-capacity would free up space for executive schemes and be more efficient in complex visual search tasks under higher working memory load situations. Thus, if higher intelligence quotient (IQ) is related to better Mental Attention performance, would children with higher IQ levels also be more efficient in visual search tasks under high working memory loads? Also, metacognitive strategies are associated with better performance in cognitive tasks (Bewick et al., 1995) and better academic performance (Pintrich and de Groot, 1990; Paloş et al., 2011). During a visual search, there are two moments when participants could use metacognitive strategies: when coding the target in working memory (for instance by repetition or by mental imagery) and organizing visual tracking (looking in reading-order, up-down, left-right). However, not all children can self-generate strategies or use fewer effective strategies. It can depend on their developmental moment. For example, younger children of 4–5 years tend to prefer visual coding in working memory, while older children above 8–10 years tend to prefer verbal codes to maintain temporal information in working memory (Palmer, 2000; Henry et al., 2012). In the Model of Endogenous Mental Attention, the executive schemes are metacognitive strategies controlled by the E-operator but hyperactivated by the M-operator.

This study aims to understand visual search modulations under different working memory load conditions in a dual-task paradigm in a developmental study. To our knowledge, we are the first to study these interactions under those classic dual-task paradigms. We will use the Pirate-Treasure visual search task designed by Gil-Gómez de Liaño et al. (2020) in a sample of kindergarten to college students. Observers must look for real-world photorealistic targets while maintaining similar objects in visual working memory. We will refer to working memory without specifying the kind of content stored because the Model of Endogenous Mental Attention describes the operators as content-free. We will manipulate working memory load by including two memory load conditions: high load with four images to remember and low load with one. We will measure accuracy, reaction times, search slopes, and efficiency in the visual search task to find differences under different memory load conditions. Although in our experimental design we would not expect to find differences in adults considering the evidence in the field, we expect to find a larger impairment in visual search for the youngest children, especially under high load conditions, as both attentional control and working memory capacity are still in maturation. In terms of the Model of Endogenous Mental Attention, if the I-operator is not yet developed in the early developmental stages to deactivate the schemes of the concurrent working memory task, there should be a decrease in accuracy and a general decrement in efficiency performing the visual search task. Also, we will control and study how other secondary potentially related individual differences in intelligence, executive functions, and working memory span can affect visual search efficiency. We expect to find a positive correlation between visual search efficiency and intelligence, working memory span, and executive function capacity. Finally, we will ask the participants about the type of strategy followed during the task to understand how metacognitive strategies could affect potential changes in visual search performance and whether they could be related to age strategy changes during development. We will discuss the implications in educational contexts.

## Materials and methods

### Participants

**FIGURE 2:**
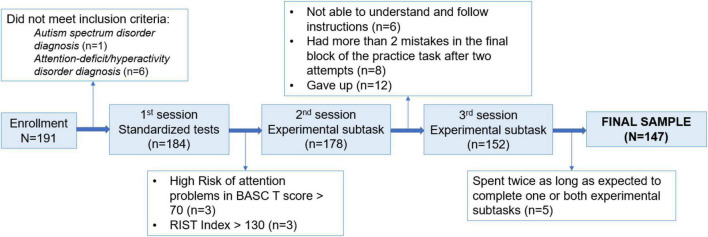
Flow of participants in each stage of the study. CPT, Conners Continuous Performance Test; K-CPT, Conners Kiddie Continuous Performance Test; and BASC, Parent Report form of The Behavioral Assessment Scale for Children.

We recruited 191 participants from public and private elementary and middle schools in Madrid, Spain, and college students from Universidad Autónoma de Madrid. From previous studies on the lifespan in visual search (Gil-Gómez de Liaño et al., 2020), looking for age differences showed that with the alpha set to 0.05 and 1-beta (power) of over 0.9, we could detect significant effects (partial eta-square η^2^ = 0.01) if we run between 21 and 33 participants per group of age. Thus, our sample size allowed for sizeable cohorts in each age, with a minimum of 21 for each age group (refer to Table 1). After missing several participants for different reasons explained in Figure 2, the final sample was composed of 115 children divided into four groups at different school levels and 32 psychology undergrad students (refer to Table 1 for sociodemographic descriptive statistics).

**TABLE 1:** Sociodemographic descriptive statistics of the final sample for age and IQ.

			Age				IQ			
Grade		*n*	*M*	*SD*	Min	Max	*M*	*SD*	Min	Max
Kindergarten	TOTAL	25	5.6	0.5	5	6	104.42	11.11	84	129
	Male	11	5.64	0.505	5	6	102.18	7.92	84	110
	Female	14	5.57	0.51	5	6	106.31	13.26	88	129
2nd elementary	TOTAL	28	7.14	0.36	7	8	109.86	10.45	77	122
	Male	16	7.06	0.25	7	8	109.81	13.03	77	121
	Female	12	7.25	0.452	7	8	109.92	6.05	101	122
4th elementary	TOTAL	31	9.16	0.37	9	10	109.65	11.94	74	130
	Male	13	9	0	9	9	111.77	11.25	91	130
	Female	18	9.28	0.46	9	10	108.11	12.49	74	125
6th middle school	TOTAL	31	10.97	0.41	10	12	104.9	10.02	83	122
	Male	8	10.88	0.35	10	11	108.75	10.91	88	122
	Female	23	11	0.43	10	12	103.5	9.56	83	121
College students	TOTAL	32	19.78	1.84	18	25	102.09	12.37	77	129
	Male	11	19.91	2.39	18	25	103.73	14.14	77	129
	Female	21	19.71	1.55	18	25	101.24	11.61	79	123

M, mean; SD, standard deviation; Min, minimum; Max, maximum; IQ, intelligence quotient measured by Reynolds Intellectual Screening Test (RIST).

All the participants had a normal or corrected-to-normal vision and no history of neurological, motor impairment, or generalized developmental disorders. They gave verbal and/or written assent and written informed consent signed by parents or guardians in the case of minors. The children got “Pirate” diplomas as a reward, and the college students got credits for courses. The study was approved in advance by the ethics committee of Universidad Autónoma de Madrid (CEI-84-1553).

### Instruments and procedure

Each participant completed three sessions, the children at schools and the college students at the university. We applied several standardized tests to eliminate potentially non-typical developing individuals in the first session and control and study potential effects based on individual differences.

#### Session 1: The standardized tests

Intelligence quotient (IQ) was measured by *Reynolds Intellectual Screening Test* (RIST, Reynolds and Kamphaus, 2003). RIST includes a verbal subtest (*Guess What*, crystallized intelligence screening) and a non-verbal subtest (*Odd-Item Out*, fluid intelligence screening), and it takes between 20 and 30 min to complete.

We assessed visual working memory by the *Picture Span* subtest from *Wechsler Intelligence Scale for Children 5th Edition* (WISC-V, Spanish edition; Wechsler et al., 2015). The participants had to remember a set of pictures to recognize later in the same order with difficulty increasing in each trial. Standardized scores are only available for children from 6 to 16 years old. Therefore, we used the eldest scale range (16 years and 11 months) for the young adults’ sample.

Parents filled out two standardized questionnaires for the children: first, the *Parent Report* form of *The Behavioral Assessment Scale for Children* (BASC; Reynolds and Kamphaus, 2004) measures adaptive and behavioral problems in the community and at-home settings. The test can identify and differentiate between attention problems and hyperactivity. Second, we used the *Parent Report* of *The Behavior Rating Inventory of Executive Function* (BRIEF; Gioia et al., 2015), which detects potential difficulties in executive functions at home and school.

Finally, the young adults and parents filled out a short questionnaire about the participants’ basic development and medical background.

#### Sessions 2 and 3: The experimental tasks

The experiment was coded using *E-prime 3.0* (Psychology Software Tools Inc., 2016). Stimuli were 2,469 real-word images provided by Brady et al. (2008). We divided the images into four sets: two for the visual search (targets and distractors pool of images) and two for the working memory concurrent task (images to remember and show as distractors in the working memory probe and testing phase). We aimed to avoid any potential confounding/overlapping of the images between the tasks. Thus, any visual search image would never appear in the working memory set and vice versa. For the target set of images in the visual search task, we included only child-friendly images to increase motivation for the participants. They responded with a touch-screen computer (*Microsoft surface Pro i5*) with an 800 pixel × 600 pixel monitor resolution. Image size was 96 pixels × 96 pixels each.

The participants must follow some basic instructions before starting the task: both hands had to be at the sides of the tablet on the table, and they could only respond with their dominant hand. After every tap-answer, the hand must return to rest on the tablet’s side again. If a child was too young to follow the basic instructions, having problems inhibiting their non-dominant hand, they should put the non-dominant hand under their thighs on the chair.

The experimental task included two subtasks: a subtask with low working memory load (low-load, one image) and another subtask with high working memory load (high-load, four images). Low-load took 10–20 min, and high-load took around 20–30 min. The subtasks were counterbalanced, with half of the participants running low-load/high-load and the other half high-Load/low-load. We run them on different session days, sessions 2 and 3 of the whole testing. There were no more than 3 days between sessions.

**FIGURE 3:**
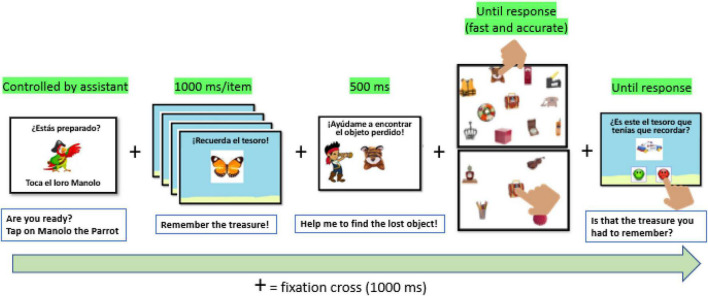
Example of a dual-task trial. In the original version, the size ratio of the images is stated in the method. The instructions were written in Spanish, the language spoken by the participants; the translation in English is included below each image.

We told the participants they were pirates with two missions: look for treasures stolen by evil pirates and remember the treasures buried on the beach to prevent further thefts. An example of a full trial is shown in Figure 3. A trial started with the picture of a parrot that remained until response (tapping anywhere on the screen) to prepare for the subsequent trial. That way, every participant could adjust the speed of the task according to the age and control they were attending to the task. Then, a white background with a centered cross remained on the screen for 1,000 ms before the beach-treasure/s (one for the low-load and four for the light-load) were presented in the beach background for the working memory maintenance task. Images for the concurrent working memory task appeared on the center of the screen one at a time for 1,000 ms, one after another. For the low-load, there was only one image lasting 1,000 ms, too. Then, the target for the visual search appeared for 500 ms in the same position (refer to Figure 3). Right after, the search display appeared.

The participants had to look for the target among distractors and respond as quickly and accurately as possible. Set size could be 8 or 32 items, including the target, randomly presented. Half of the trials contained the target, and the other half did not. They were presented in random order. Thus, there were 7 distractors plus the target for the target-present trials, while 8 items were distractors for the target-absent trials. The same rationale was applied for set size 32. The target position for each trial was also randomized. If the target was present, the participants must tap on it. For the target-absent trials, they had to tap on the pirate chest in the center of the screen (which appeared in all the trials regardless if the target was or was not present). Thus, it was both an identification and a localization visual search task. Search displays remained on the screen until a response. Finally, the beach background appeared again for the working memory test. The test consisted of an image that was the one shown in the low-load condition or one of those shown in the high-load condition for half of the test trials. The other half contained a different image randomly selected from the working memory pool. If the testing image was one of the high-load or the low-load images, the observer must tap on a green smiling face shown at the bottom of the screen, while if it was not, they must tap on the sad red face also at the bottom (refer again to Figure 3). The order of green/red correct responses was again randomized. The beach-test display remained on the screen until a response with no emphasis on speeded responses as in the visual search task.

The final design included set size (8/32), target (present/absent), and working memory load (low-load/high-load) as within-subjects factors, while grade was the between-subject factor (kindergarten, KG; 2nd elementary, 2nd; 4th elementary, 4th; 6th middle school, 6th; and college students, college). Each experimental subtask in sessions 2 and 3 consisted of 120 trials, with 240 for the whole task. Every 40 trials, a “progress bar” showed up as a reinforcement to keep the participants motivated throughout the task. A congratulatory message for recovering all the pirate treasures was also displayed at the end of each subtask. The participants initially performed a 24-trial practice block with feedback and the experimenter present to make sure they understood the task for every subtask. There was no feedback in the experimental blocks.

At the end of each experimental session, we asked the participants about the cognitive strategies potentially used during the visual search. First, we asked them if they used any “tricks” to remember the objects in each trial (potential maintenance strategies). We asked them to choose between using “no trick; look and that’s it,” no strategy, a perceptual strategy (“color,” “shape,” or “picture”), and a verbal strategy (“naming/repeating the object out loud or in your mind”). Then, we asked them an open-response question, if they had used any “trick” to search faster (looking for potential visual tracking strategies). Because of the small number of participants reporting using tracking strategies (e.g., searching from left to right or up-down), we divided them into those using them and those not using them for the analysis.

## Data analysis

The analyses were conducted using R version 4.0.3 (R Core Team, 2020) and SPSS Statistics 26 (IBM Corp, 2019).

### Visual search performance

Trials with null responses in the visual search or the working memory task, that is, when the participants tapped anywhere outside the response areas (the images), were eliminated (<0.01%). In the case of the visual search task, trials with responses shorter than 200 ms and longer than 9,000 ms were also eliminated (<0.04% of the data), following Gil-Gómez de Liaño et al. (2020)’s criteria. We performed visual search data analysis only for trials with a correct response in the working memory task (85% of the trials) to ensure that our working memory manipulation effectively loaded memory during the search. The results were similar when all the trials were included (also working memory task errors). To ensure that the manipulation of the working memory task has been sufficient to cause two different load levels, interested readers can find the working memory accuracy analysis in the Supplementary Material.

For each condition of the visual search task, we collected latency (reaction times, RTs) and accuracy measures (correct responses; false alarms, tapping a distractor when the target is absent; commissions errors, tapping a distractor when the target is present; omissions errors, tapping the pirate chest when the target is present). Note that like in Gil-Gómez de Liaño et al. (2020), the design of our experiment allows for us to analyze commission errors when the target is present since the observers must localize the target. Thus, the observers can identify it as a non-target and tap instead on a distractor identified as the target in those error trials. We used the raw data for correct responses, false alarms, commissions, and omissions, analyzing them as a dichotomous dependent variable (yes/no response). Mean RTs (in milliseconds, ms) and search slopes were calculated for each participant’s different combinations of variables (refer later on the use of General Linear Mixed Models, GLMMs).

In addition to the latency and accuracy measures in visual search, we calculated an efficiency measure that controlled the trade-off between speed and accuracy that usually occurs in visual search tasks: the observers adjust their response speed to make fewer mistakes based on the task’s difficulty. The Inverse Efficiency Score (Townsend and Ashby, 1983) is one of the most widespread indices. However, we need to meet two assumptions: the proportion of correct responses must be greater than 0.90 (Bruyer and Brysbaert, 2011), and there must be a strong positive linear correlation between RT and the proportion of errors (Townsend and Ashby, 1983). According to the present study’s data, we cannot assume these two assumptions. As an alternative, we calculated the Efficiency Score proposed by Roncadin et al. (2007). The formula is as follows:


$$E\,f\,f\,i\,c\,e\,n\,c\,y\,S\,c\,o\,r\,e=\frac{R\,T\,\left(R\,T-100\,n\right)/R\,T\,\left(R\,T+100\,n\right)}{R\,T\,\left(R\,T-P\,C\,n\right)/R\,T\,\left(R\,T+P\,C\,n\right)}$$

where *RT* is the individual’s mean reaction time, *PC* is the individual’s mean proportion of correct responses, and *n* is the sample’s minimum RT divided by twice the sample’s maximum proportion of correct responses. The minimum RT for the present sample was 706.34 ms, and the maximum proportion was 100 (*n* = 3.532). The distribution of the Efficiency Score falls between 0 (worst performance, the proportion of correct responses is 0) and 1 (perfect performance). Thus, higher scores reflect better efficiency regardless of observers’ response styles.

We conducted accuracy analyses using GLMM for the log-odds ratio of correct responses, false alarms, commissions, and omissions to control individual variability. We used linear mixed-effects models for continuous variables (RT, search slopes, and efficiency score).

The hierarchical structure of the data was analyzed under a two-level random intercept model. Independent variables were included in level 1 and organized within observers (level 2). In level 1, for efficiency score, correct responses and RT-dependent variables, working memory load, target, and set size were the independent factors. In the case of errors, the factors were working memory load and set size. Finally, working memory load and target were the independent variables for the search slope analysis. In all the analyses, grade was added as a predictor to level 2 to study the developmental effect, which is one of our main objectives of the present study.

The estimation of fixed regression coefficients was based on maximum likelihood to allow for model comparisons (Field et al., 2012). We conducted Hommel corrections (Hommel, 1988) for *post hoc* comparisons between conditions in the case of the linear mixed-effects models. For comparisons in the GLMM, and due to the difficulty usually found in interpreting the odds ratios in this type of model, we used plots that include their transformation to estimated proportions and the confidence intervals of each estimation, as recommended by Gelman and Hill (2007).

To build the final models, we started from the null model, not including parameters, in which the intraclass correlation coefficients (ICCs) were between 0.017 and 0.33. This verifies the individual variability among the participants and the pertinence to use such models instead of conducting regular ANOVAs. We added to the null model the parameters, main effects as well as interactions, one by one to the null model, testing the fit to the data and the improvement of the model in each step by comparing changes in the log-likelihood between models, as well as Akaike’s information criterion (AIC) and Schwarz’s Bayesian Criterion (BIC). Parameters that did not significantly improve the fit were discarded. For interested readers, the building progression of the final models is shown in Supplementary Material, including comparisons with the null model and the maximal model with all the parameters included.

### Individual differences and visual search efficiency

To study the relationship between individual differences and efficiency in visual search, we conducted a partial correlation analysis controlling for age (in months) to eliminate developmental effects. Specifically, we correlated the average efficiency score in each of the eight conditions resulting from combining the three within-subjects experimental factors in visual search (working memory load, set size, and target) with the RIST T-Scores (both for the two subtests as well as for the general index), the scalar scores provided by the *Picture span* test from WISC-V, and the BRIEF and BASC *T*-Scores. Correlations between ES and BRIEF, BASC, and *Picture span* were only analyzed for the children since the tests were not scaled for the population over 17 years of age.

### Metacognitive strategies change

In the working memory low-load session, 9 participants did not answer the memory strategy question, and 7 did not answer the visual tracking one. In the working memory high-load condition, they were 13 participants who did not answer the memory strategy question and 11 who did not respond to the visual tracking question. We did not consider these participants for the final analysis in the study on metacognitive strategies. We performed chi-square tests of independence to assess whether memory and visual tracking strategy changes were related to the different age groups. We calculated Cramer’s *V* to measure effect size.

## Results

### Visual search performance

**FIGURE 4:**
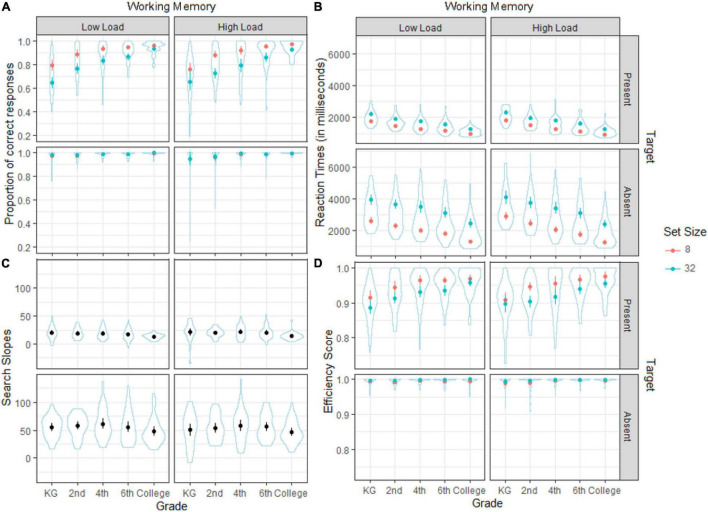
Mean proportion of **(A)** correct responses, **(B)** reaction time, **(C)** search slopes, and **(D)** efficiency score as a function of grade, set size, working memory load, and target. Mean of search slopes as a function of grade, working memory load, and target. Bars represent the confidence intervals for each mean represented by dots.

In Figure 4, we can see the mean proportion of correct responses (A), reaction times (B), search slopes (C), and efficiency score (D) for the visual search task as a function of the target (present/absent), working memory load (low-load/high-load), set size (8–32, except for search slopes), and grade (KG to college).

#### The M-operator and the I-operator functioning: accuracy, false alarms, commissions, and omissions

As we can see in the upper panels of Figure 4A (target-present conditions), the proportion of correct responses in the visual search task seems higher for the low-load than for the high-load, as corroborated by the significant effect of working memory shown in the GLMM analysis in Table 2. The probability of a hit in the visual search is higher for the low-load condition. However, interactions between working memory and target or set size were not significant (refer again to Table 2).

**TABLE 2:** Estimated coefficients for correct responses (odds ratios) in the visual search task.

Fixed effects – predictors	OR	SE	CI	*t*	*p*
Intercept (KG, working memory low load, target present, set size 8)	4.08	0.55	3.13 – 5.30	10.47	**<0.001**
Set size (32)	0.48	0.05	0.40 – 0.58	−7.58	**<0.001**
Target (absent)	7.90	1.24	5.81 – 10.74	13.17	**<0.001**
Working memory (high load)	0.89	0.04	0.81 – 0.98	−2.36	**0.018**
Grade (2nd)	2.19	0.41	1.52 – 3.16	4.20	**<0.001**
Grade (4th)	4.07	0.78	2.79 – 5.92	7.33	**<0.001**
Grade (6th)	5.42	1.07	3.68 – 7.96	8.59	**<0.001**
Grade (college)	7.71	1.58	5.16 – 11.53	9.95	**<0.001**
Setsize (32) × Target (absent)	3.85	0.59	2.85 – 5.20	8.78	**<0.001**
Target (absent) × Grade (2nd)	0.53	0.10	0.36 – 0.77	−3.26	**0.001**
Target (absent) × Grade (4th)	0.93	0.22	0.58 – 1.49	−0.31	0.754
Target (absent) × Grade (6th)	0.62	0.15	0.39 – 0.99	−2.01	**0.045**
Target (absent) × Grade (college)	0.88	0.27	0.48 – 1.60	−0.43	0.670
Set size (32) × Grade (2nd P)	0.75	0.10	0.58 – 0.99	−2.05	**0.040**
Set size (32) × Grade (4th P)	0.67	0.10	0.50 – 0.90	−2.64	**0.008**
Set size (32) × Grade (6th P)	0.70	0.11	0.51 – 0.95	−2.26	**0.024**
Set size (32) × Grade (college)	1.01	0.18	0.71 – 1.43	0.07	0.946
**Random effects**					
σ^2^	3.29				
τ_00 *Subject*_	0.29				
Intraclass correlation coefficient	0.08				
Marginal *R*^2^/Conditional *R*^2^	0.376/0.427				

OR, odds ratio; SE, standard error; CI, confidence interval. Refer to the building progression of the model in Supplementary Material. The values indicate that *p* < 0.05 and therefore, the effect to which they refer is significant.

**FIGURE 5:**
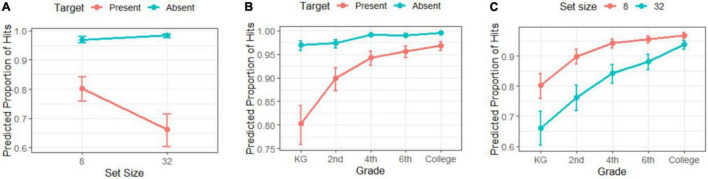
Proportion of correct responses estimated with the General Linear Mixed Model in the visual search task as a function of **(A)** set size and target, **(B)** grade and target, and **(C)** set size and target. The bars represent CIs for each estimation (dots).

Regarding the rest of the factors, the main effect of target was significant: The likelihood of a correct response was higher when the target is absent (showing a ceiling effect on all grades regardless of set size, as shown in Figure 4A). The main effect of set size was also significant, showing worse accuracy as distractors increase, although this occurred only when the target was present (refer to confidence intervals in Figure 5A).

For grade, as expected the significant main effect indicated an increase in accuracy with age. Its interaction with target was also significant (refer again to Table 2): for the target-present conditions, there was a gradual improvement in correct responses with age, especially pronounced at younger ages, while for target-absent the ceiling effects appeared from 4th grade onward (although even younger children showed very high accuracy; see confidence intervals in Figure 5B). There was also a main effect for the interaction between set size and grade (refer to Table 2). Changes between grades are smoother with fewer distractors (set size 8) from 4th grade observers onward, while under search conditions with more distractors (set size 32), there are still significant changes in the higher grades (see confidence intervals in Figure 5C). Also, the difference in accuracy between set size 8 and 32 was minor in college observers compared to the rest of grades. Although with minor changes, these results essentially replicate those found in Gil-Gómez de Liaño et al. (2020) under no working memory load conditions.

To study if the effect of working memory load that we found in accuracy affects the M-operator and the I-operator in the same way, we analyzed the error patterns separately. Note that false alarms and commissions are related to failure of the I-operator to deactivate schemes irrelevant to the task, while omissions would be due to lack of target hyper-activation by the M-operator. For false alarms and commissions, as we can see in Table 3, the probability of committing those types of errors was higher when working memory is highly loaded and as grade decreases. However, the main effect of grade was not affected by working memory (not significant interaction), showing that the likelihood of showing these errors was lower with age regardless of memory load. The effect of set size was only significant for the false alarms: it was more likely to have a false alarm with 32 objects on the screen than with 8 objects.

**TABLE 3:** Estimated coefficients for false alarms, commissions, and omissions (odds ratios) in the visual search task.

	False alarms					Commissions					Omissions				
Fixed effects – predictors	*OR*	*SE*	CI	*t*	*p*	*OR*	SE	CI	*t*	*p*	*OR*	SE	CI	*t*	*p*
Intercept	0.02	0.00	0.01 – 0.03	−14.13	**<0.001**	0.00	0.00	0.00 – 0.01	−11.69	**<0.001**	0.24	0.03	0.19 – 0.32	−10.40	**<0.001**
Working memory (high load)	1.38	0.20	1.04 – 1.84	2.25	**0.025**	2.49	0.63	1.52 – 4.07	3.63	**<0.001**					
Set size (32)	0.69	0.10	0.52 – 0.91	−2.57	**0.010**						1.97	0.20	1.61 – 2.40	6.58	**<0.001**
Grade (2nd)	0.99	0.35	0.49 – 2.00	−0.03	0.978	0.59	0.35	0.18 – 1.91	−0.88	0.380	0.44	0.09	0.30 – 0.65	−4.21	**<0.001**
Grade (4th)	0.35	0.13	0.16 – 0.73	−2.77	**0.006**	0.36	0.22	0.11 – 1.19	−1.67	0.096	0.24	0.05	0.16 – 0.35	−7.27	**<0.001**
Grade (6th)	0.35	0.13	0.17 – 0.75	−2.72	**0.006**	0.27	0.17	0.08 – 0.93	−2.08	**0.038**	0.19	0.04	0.12 – 0.28	−8.22	**<0.001**
Grade (college)	0.16	0.07	0.07 – 0.36	−4.37	**<0.001**	0.07	0.06	0.02 – 0.35	−3.28	**0.001**	0.13	0.03	0.08 – 0.19	−9.71	**<0.001**
Set size (32) × Grade (2nd)											1.46	0.22	1.09 – 1.97	2.52	**0.012**
Set size (32) × Grade (4th)											1.69	0.27	1.23 – 2.31	3.25	**0.001**
Set size (32) × Grade (6th)											1.55	0.26	1.11 – 2.16	2.55	**0.011**
Set size (32) × Grade (college)											1.17	0.22	0.81 – 1.70	0.82	0.410
**Random effects**															
σ^2^	3.29					3.29					3.29				
τ_00 *Subject*_	1.01					2.29					0.30				
Intraclass correlation coefficient	0.24					0.41					0.08				
Marginal *R*^2^/Conditional *R*^2^	0.112/0.321					0.147/0.497					0.160/0.231				

OR = odds ratio, SE = standard error, CI = confidence interval. Intercepts for omissions were KG and set size 8 and for false alarms and commissions KG and low working memory load. The rows without data correspond to parameters that did not improve the models and were not included. Refer to the building progression of the models in Supplementary Material. The values indicate that *p* < 0.05 and therefore, the effect to which they refer is significant.

**FIGURE 6:**
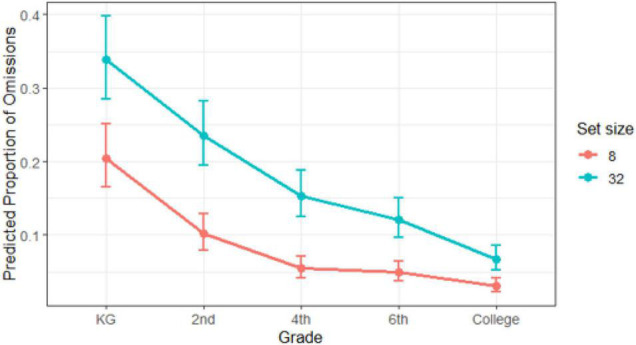
Proportion of omissions estimated with the General Linear Mixed Model in the visual search task as a function of grade. Bars represent confidence intervals for each estimation (dots).

For omissions, however, the working memory load did not reach significance in the final model (refer to Table 3), nor did the interactions with set size and grade. Grade was significant, though, showing that the probability of omissions decreased with age. Nonetheless, differences were minimal for older children from the 4th grade onward for set size 8, as we can see comparing the confidence intervals in Figure 6, while the younger ones showed more pronounced effects between them and the rest. The probability of missing a target was higher when the search display was 32 for all the groups (refer again to Table 3 and Figure 6).

#### The E-operator functioning: reaction times and search slopes

As we can see in Table 4, the main effect of working memory load is again significant, with larger RTs under high-load conditions and in the same way as the proportion of correct responses. However, there were no interactions between working memory load and the rest of the visual search factors (target or set size) or a triple or quadruple interaction among all the factors. However, the interaction between working, memory load and grade showed a tendency for the youngest children (KG and 2nd) with higher RTs for high-load conditions compared to the older ones. *Post hoc* comparisons with no corrections showed significant differences between load conditions for KG (*p* = 0.007) and 2nd (*p* = 0.056), with these younger children spending more time when the target was present in the high-load condition. Indeed, if we consider RT differences between both levels in working memory load in different ages, KG obtained significant RT differences compared to the rest of the groups, except for the 2nd grade (refer to Table 4 again). There were no differences among the rest of the groups.

**TABLE 4:** Estimated coefficients for reaction times in the visual search task.

Fixed effects – predictors	Estimates	*SE*	CI	*t*	*p*
Intercept (KG, working memory low load, target present, set size 8)	1738.10	96.98	1548.02 – 1928.17	17.92	**<0.001**
Set size (32)	446.65	60.21	328.64 – 564.66	7.42	**<0.001**
Target (absent)	909.94	60.21	791.92 – 1027.95	15.11	**<0.001**
Working memory (high load)	150.74	55.66	41.64 – 259.84	2.71	**0.007**
Grade (2nd)	−305.11	132.49	−564.78 – −45.43	−2.30	**0.021**
Grade (4th)	−466.60	129.43	−720.27 – −212.92	−3.61	**<0.001**
Grade (6th)	−564.17	129.43	−817.85 – −310.50	−4.36	**<0.001**
Grade (college)	−769.49	128.52	−1021.39 – −517.59	−5.99	**<0.001**
Set size (32) × Target (absent)	855.82	45.91	765.84 – 945.81	18.64	**<0.001**
Set size (32) × Grade (2nd)	28.32	76.58	−121.78 – 178.41	0.37	0.712
Set size (32) × Grade (4th)	80.62	74.81	−66.01 – 227.26	1.08	0.281
Set size (32) × Grade (6th)	16.97	74.81	−129.66 – 163.60	0.23	0.821
Set size (32) × Grade (college)	−143.32	74.29	−288.93 – 2.29	−1.93	0.054
Target (absent) × Grade (2nd)	−21.59	76.58	−171.69 – 128.50	−0.28	0.778
Target (absent) × Grade (4th)	−144.26	74.81	−290.89 – 2.38	−1.93	0.054
Target (absent) × Grade (6th)	−302.07	74.81	−448.70 – −155.44	−4.04	**<0.001**
Target (absent) × Grade (college)	−600.08	74.29	−745.69 – −454.48	−8.08	**<0.001**
Working memory (high) × Grade (2nd)	−50.77	76.58	−200.87 – 99.33	−0.66	0.507
Working memory (high) × Grade (4th)	−151.04	74.81	−297.67 – −4.41	−2.02	**0.044**
Working memory (high) × Grade (6th)	−180.39	74.81	−327.02 – −33.76	−2.41	**0.016**
Working memory (high) × Grade (college)	−176.01	74.29	−321.61 – −30.40	−2.37	**0.018**
**Random effects**					
σ^2^	154923.09				
τ_00 *Subject*_	154370.24				
Intraclass correlation coefficient	0.50				
Marginal *R*^2^/Conditional *R*^2^	0.702/0.851				

SE, standard error; CI, confidence interval. Refer to the building progression of the model in Supplementary Material. The values indicate that *p* < 0.05 and therefore, the effect to which they refer is significant.

Again, as expected from previous studies, RTs were larger for set size 32 and target-absent conditions (refer to Table 4). The interaction was also significant: set size effects were larger under target-absent conditions, replicating previous results again (Gil-Gómez de Liaño et al., 2020).

As for the correct responses, the effect of grade produced a gradual decrease in RT. The difference was significant even for the 6th graders and college students, as shown in Table 5, in the *post hoc* comparisons.

**TABLE 5:** *Post hoc* comparisons for RT (in ms) in the visual search task between different grade levels under different target and set size conditions.

Comparison	Target present		Target absent		Set size 8		Set size 32	
	Mean difference	*P*-value	Mean difference	*P*-value	Mean difference	*P*-value	Mean difference	*P*-value
KG – 2nd	316	0.054	338	**0.03**	341.3	0.052	313	0.08
KG – 4th	502	**<0.001**	646	**<0.0001**	614.2	**<0.0001**	533.6	**<0.001**
KG – 6th	646	**<0.0001**	948	**<0.0001**	805.4	**<0.0001**	788.4	**<0.0001**
KG – college	929	**<0.0001**	1529	**<0.0001**	1157.5	**<0.0001**	1300.9	**<0.0001**
2nd – 4th	185	0.28	308	**0.04**	273	0.12	220.6	0.19
2nd – 6th	330	**<0.05**	610	**<0.0001**	464.1	**<0.01**	475.5	**<0.01**
2nd – college	613	**<0.0001**	1191	**<0.0001**	816.2	**<0.0001**	987.9	**<0.0001**
4th – 6th	144	0.28	302	**0.042**	191.2	0.28	254.8	0.15
4th – college	427	**<0.01**	883	**<0.0001**	543.3	**0.0001**	767.2	**<0.0001**
6th – college	283	0.06	581	**0.0001**	352.1	**0.02**	512.4	**<0.001**

The values indicate that *p* < 0.05 and therefore, the effect to which they refer is significant.

Interestingly, the differences in RT between set sizes 8 and 32 were significantly lower for the college students that for the rest of the grades (β = 142.3, *p* = 0.054 for KG; β = 171.6, *p* = 0.017 for 2nd; β = 223.9, *p* = 0.001 for 4th; β = 160.3, *p* = 0. 022 for 6th). There were no differences among all the children, indicating that the interference due to increase in the distraction (set size manipulation) was indeed lower for the young adults than for the rest of the observers in the sample. This effect for older children in the 4th and 6th grades (9–12 years old) was not found in Gil-Gómez de Liaño et al. (2020). Although we have not seen an interaction between working memory load and set size for the proportion of correct responses or RTs, maybe the presence of a concurrent working memory task has made a difference.

Finally, we analyzed the search slopes to differentiate whether the effect of memory load was caused by a problem in the E-operator or it was related to the processing speed. In this case, only the main effect of target was significant in the final model (refer to Table 6): slopes were steeper when the target was absent, indicating that the participants spent more time per item searching under these conditions, again replicating previous results in visual search with children (Gil-Gómez de Liaño et al., 2020), with no effect of working memory load.

**TABLE 6:** Estimated coefficients for search slopes in the visual searchtask.

Fixed effects – predictors	Estimates	*SE*	CI	*t*	*p*
Intercept (target present)	18.39	1.29	15.86 – 20.93	14.23	**<0.001**
Target (absent)	35.66	1.20	33.3 – 38.01	29.68	**<0.001**
**Random effects**					
σ^2^	212.17				
τ_00 *Subject*_	139.64				
Intraclass correlation coefficient	0.40				
Marginal *R*^2^/Conditional *R*^2^	0.475/0.683				

SE, standard error; CI, confidence interval. Refer to the building progression of the model in Supplementary Material. The values indicate that *p* < 0.05 and therefore, the effect to which they refer is significant.

#### Global functioning of the M, I and E-operators: efficiency score

We calculated the efficiency score (Roncadin et al., 2007) to determine to what extent the coordinated functioning of the M-, I, and E-operators might be affected by working memory load in visual search. The final model for efficiency score showed essentially significant main effects for grade, target, and set size (refer to Table 7). However, working memory load was not a significant parameter, neither were any of the interactions with the rest of the variables: Working memory load did not affect the visual search task’s efficiency.

**TABLE 7:** Estimated coefficients for efficiency score in the visual search task.

Fixed effects – predictors	Estimates	*SE*	CI	*t*	*p*
Intercept (KG, target present, set size 8)	0.92	0.00	0.91 – 0.92	224.59	**<0.001**
Set size (32)	−0.03	0.00	−0.03 – −0.02	−11.26	**<0.001**
Target (absent)	0.08	0.00	0.07 – 0.08	16.96	**<0.001**
Grade (2nd)	0.02	0.01	0.01 – 0.04	4.60	**<0.001**
Grade (4th)	0.04	0.01	0.03 – 0.05	7.69	**<0.001**
Grade (6th)	0.05	0.01	0.04 – 0.06	9.44	**<0.001**
Grade (college)	0.06	0.01	0.05 – 0.07	11.92	**<0.001**
Setsize (32) × Target (absent)	0.03	0.00	0.02 – 0.04	8.95	**<0.001**
Target (absent) × Grade (2nd)	−0.02	0.01	−0.04 – −0.01	−4.28	**<0.001**
Target (absent) × Grade (4th)	−0.04	0.01	−0.05 – −0.03	−6.54	**<0.001**
Target (absent) × Grade (6th)	−0.05	0.01	−0.06 – −0.03	−8.22	**<0.001**
Target (absent) × Grade (college)	−0.06	0.01	−0.07 – −0.05	−10.37	**<0.001**
**Random effects**					
σ^2^	0.00086				
τ_00 *Subject*_	0.00016				
Intraclass correlation coefficient	0.16				
Marginal *R*^2^/Conditional *R*^2^	0.523/0.599				

SE, standard error; CI, confidence interval. Neither working memory nor any of its interactions significantly improved the fit of the model, and they were not included. Refer to the building progression of the model in Supplementary Material. The values indicate that *p* < 0.05 and therefore, the effect to which they refer is significant.

As expected, for set size 32, efficiency was lower, while for target-absent conditions, efficiency was higher (refer to the main effect in Table 7). However, the differences between set size conditions were only significant under target-present conditions (*p* < 0.0001) and disappeared under target-absent ones (*p* = 0.165). Finally, although there was a significant effect of grade with higher efficiency as age increases, it only arose again for target-present conditions: the KG children achieved the lowest efficiency followed by the 2nd grade children (*p* = <0.0001, for all the comparisons with the other grades and between them, except between 2nd and 4th where *p* = 0.034). There were no efficiency differences between 4th and 6th (*p* = 0.755) or between 6th and the College students (*p* = 0.15). However, the 4th-grade children were less efficient than the college group (*p* ≤ 0.001).

### Individual differences and visual search efficiency

We found significant positive correlations between the RIST and the *Picture span* tests with efficiency in the visual search task, but only when the target was present (refer to Table 8). The RIST general index was positively correlated with the remaining four efficiency measures: the higher the capacity (IQ), the higher the efficiency. Specifically, the high scores in the *Odd-Item Out* subtest were related to higher efficiency under low-load conditions. For the *Guess What* subtest, the higher scores were also related to higher efficiency but only for high-load conditions, and for the low-load under set size 8. *Picture span* scalar score was only correlated with efficiency in high-load for set size 8. Finally, we did not find significant correlations between efficiency score and BRIEF and BASC *T*-scores.

**TABLE 8:** Partial correlations matrix (controlling for age in months) between efficiency scores in each of the eight conditions resulting from combining the 3 experimental variables in visual Search (working memory load, set size, and target) with *T*-scores from RIST and scalar scores from Picture Span (WISC-V).

		Efficiency score							
	
		LL,TP, S8	LL, TP, S32	LL, TA, S8	LL, TA, S32	HL,TP, S8	HL, TP, S32	HL, TA, S8	HL, TA, S32
Guess what – RIST subtest	Pearson’s *r*	**0.213**	0.131	0.051	0.024	**0.223**	**0.208**	−0.04	0.04
	*p*−value	**0.01**	0.119	0.544	0.774	**0.007**	**0.012**	0.632	0.632
Odd-item out – RIST subtest	Pearson’s *r*	**0.315**	**0.213**	−0.036	0.038	0.119	**0.226**	−0.028	−0.016
	*p*-value	**<0.0001**	**0.011**	0.665	0.648	0.157	**0.006**	0.74	0.85
RIST general index	Pearson’s *r*	**0.327**	**0.205**	0.021	0.04	**0.228**	**0.27**	−0.037	0.021
	*p*-value	**<0.0001**	**0.014**	0.805	0.632	**0.006**	**0.001**	0.661	0.798
Picture span – WISC-V	Pearson’s *r*	0.159	0.119	0.024	−0.095	**0.228**	0.142	0.018	0.08
	*p*-value	0.095	0.212	0.802	0.318	**0.015**	0.135	0.849	0.401

LL, low working memory load; HL, high working memory load; TP, target present; TA, target absent; S8, set size 8; S32, set size 32. Correlations controlled by age (in months). The values indicate that *p* < 0.05 and the accompanying correlation values are significant. Therefore, the effect to which they refer is significant.

### Metacognitive strategies change

**FIGURE 7:**
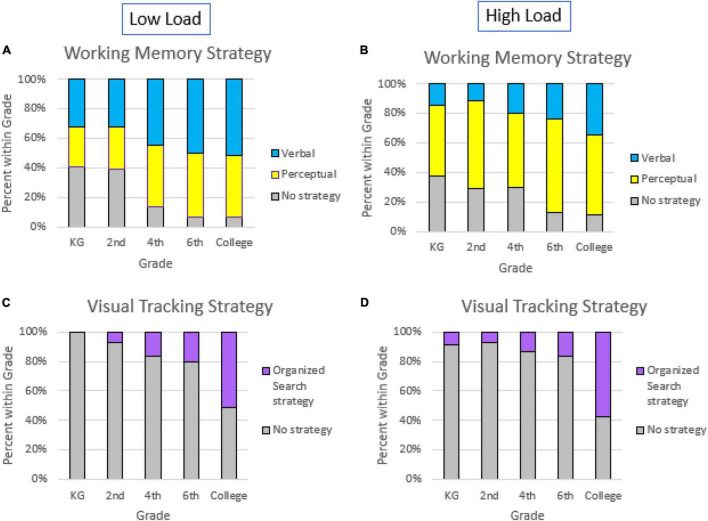
Percentage of participants who used working memory strategies to encode the target by grade in low-load **(A)** and high-load sessions **(B)**, and percentage of participants who used visual tracking strategies during the visual search by grade in low-load **(C)** and high-load **(D)** sessions.

Figures 7A,B shows the percentage of participants in each age group who used strategies during target encoding in visual search in each session (low-load and high-load). While the percentage of participants that fell into each of the three memory strategy categories (no strategy, perceptual, or verbal) did not significantly differ by group of age in the high-load session, χ^2^(8, *N* = 134) = 10.6, *p* = 0.225, they did differ for the low-load, χ^2^(8, *N* = 138) = 19.53, *p* = 0.012, *V* = 0.27. As shown in Figure 7A, the percentage of participants who did not use strategies decreased with age, especially for the 6th and college groups.

Considering the visual tracking strategies, the relationship between the age groups and the use of an organized search strategy was significant, both in the low-load session [χ^2^(4, *N* = 140) = 26.99, *p* > 0.0001, *V* = 0.44] and in the high-load session [χ^2^(4, *N* = 136) = 28.02, *p* > 0.0001, *V* = 0.45]. As shown in Figures 7C,D, the trend is similar for both working memory conditions: The percentage of participants who used tracking strategies increased with age. There was only a small percentage of children who used these strategies, while in the college students, the percentage was close to 50%.

## Discussion

Visual search is present in many daily activities at all educational levels. They are often not performed in isolation, and working memory plays a critical role in most visual search tasks. This study aimed to better understand working memory modulations in visual search from a developmental perspective based on the Model of Endogenous Mental Attention, which is part of the Theory of Constructive Operators (Pascual-Leone and Johnson, 2005, 2021). Our study is the first cross-sectional one from childhood to adulthood investigating the effects of a concurrent memory load in a dual-task paradigm in visual search from a developmental perspective. Since to our knowledge this is the first study manipulating how working memory load can modulate visual search during childhood, we avoided any potential overlapping of images between working memory and visual search tasks. Under these circumstances, our results essentially show that the efficiency of visual search is not affected by the concurrent working memory load at any age from kindergarten to college, replicating previous findings in adulthood (Downing and Dodds, 2004; Woodman and Luck, 2007; Quirós-Godoy et al., 2017). Surprisingly, even for our youngest children, the load of a concurrent visual working memory task did not make an essential difference in visual search efficiency. However, the different use of strategies in different educational stages, individual difference results, and lack of working memory modulation in visual search efficiency provide novel insights into understanding working memory and visual search interactions during development that we discuss by following the Theory of Constructive Operators.

According to the Model of Endogenous Mental Attention, the M-operator keeps the figurative scheme of the target hyper-activated to “scan and find” operative schemes during visual search. The pirate chest is always shown in the center of the screen in our task and remains active during the task, that is, visible on the screen, and therefore also in the Focus of Mental Attention. Thus, along with the distractors, they are perceptually available without consuming the M-Capacity. The results, mainly related to omissions, support this assumption: the M-operator was not overloaded because of a high load in working memory. That way, even our KG children have enough resources to efficiently perform the task with an M-capacity of 2+ *e*.

We did not find memory effects either on the target factor or the set size factor, replicating previous findings in adulthood again (Downing and Dodds, 2004; Woodman and Luck, 2007; Quirós-Godoy et al., 2017). The interaction between target and set size is consistent with previous developmental studies (Donnelly et al., 2007; Michael et al., 2013; Woods et al., 2013; Brennan et al., 2017; Gil-Gómez de Liaño et al., 2020). However, the lack of modulation of working memory loads in visual search efficiency does not mean that there is no cost of a concurrent task for the visual performance. We found a slight increase in the probability of committing errors, specifically false alarms, and commissions under high load conditions. Former studies have not found such results. Indeed, other research paradigms studying the effects of working memory load on visual search using dual-task paradigms essentially used detection but not localization visual search tasks with relative ceiling effects for correct responses basing the results on RTs. They could not study false alarms and commissions as we did.

The Model of Endogenous Mental Attention can account for this interesting modulation of working memory load on false alarms and commissions. Indeed, the I-operator could fail to effortfully deactivate the schemes of the working memory items during visual search in the high-load condition. These schemes are unnecessary for visual search but remain active within the focus of Mental Attention and cause interferences in the form of false alarms and commissions. However, we must be cautious since the percentage of these errors was very small (less than 0.012% of all the observations), leading to a potential lack of power. Actually, we found no age-group modulations, probably as we did not have errors enough to find reliable differences for the groups. However, all together, they show sufficient power to lead to significance when analyzed for the whole sample. These results are consistent with previous studies on effortful cognitive inhibition (Howard et al., 2014). This inhibition is related to the capacity of inactivating schemes that were activated previously. Howard et al. (2014) also found no age differences in some cases, and if there were, they could be attributed to diverse strategies used by children and adults.

Also, the results found for RTs show working memory modulations: the youngest children needed more time in high-load conditions, especially the KG group showing a tendency to spend more time looking for the target than the 2nd-grade children who were less affected. The trend completely disappeared in the 4th grade. However, the RT differences might have come from speed differences in information processing rather than attentional changes, as we found no slope effects for age either. The myelination process at the neuronal level is under maturation from age 5–6 to adulthood, specifically in the prefrontal cortex (Kolb et al., 2012; Miller et al., 2012).

Many studies on visual search with children suggest that executive functions might be the base of most differences in development in those tasks (e.g., Woods et al., 2013). Looking for empirical data supporting this assumption, we tested whether better executive function performance was related to better efficiency in the visual search task regardless of age. However, we did not find significant correlations between visual search efficiency and any variable from the BASC and BRIEF questionnaires. We only found an exceptional positive correlation between visual search efficiency and performance in the working memory span from the WISC-V test, similar to Guilbert et al. (2020). Part of these insignificant effects could be due to the homogeneity of the sample. All extreme values in the neuropsychological tests were eliminated as part of the inclusion criteria, involving only typical development participants.

However, we found clear evidence of a positive relationship between visual search efficiency and IQ levels, consistent with the previous literature (Navarro et al., 2006; Howard et al., 2013). The higher the IQ, the higher the visual search efficiency but only when the target was present. The low variance results across participants could explain the lack of modulation for target-absent conditions. Although high scores on the verbal and no verbal subtests of the RIST were related to better performance, it seems more pronounced for the verbal one. Certainly, language is part of numerous cognitive processes, and it is critical in the so-called *Metacognitive Knowledge*, that is, the knowledge of higher cognitive process functioning (Flavell, 1979; Efklides, 2008). Language allows for us to describe the task we perform in terms of execution, factors that can improve or worsen its achievement, or how and when to use strategies efficiently. Indeed, Vygotsky (1962) claimed that language has a critical role in developing cognitive abilities. Then, higher verbal performance could be related to better *Metacognitive Knowledge* functioning in the visual search task.

Finally, we studied how diverse strategies might affect the consecution of dual tasks. The results show an increase in the percentage of participants who used visual-tracking organization strategies with age. While the KG children barely used visual tracking strategies, 50% of the college participants used them. Reading skills (up-down; left-right) might help develop these search strategies, consistent with the increase in using them found with age (Ólafsdóttir et al., 2021). Also, from the Model of Endogenous Mental Attention, an increase in the number of schemes that the M-operator can hyperactivate at once is related to age (Pascual-Leone and Johnson, 2005, 2021; Arsalidou et al., 2019). Therefore, older groups have a greater capacity to hyperactivate and use strategies’ executive schemes, which are closely related to the speed of information processing increasing with age to manipulate more information in the brain (Fry and Hale, 2000). More free resources release the capacity to create cognitive strategies. Note that the instructions before the experimental task did not explicitly suggest any strategy. However, the participants can generate them at will. Children from 4th grade onward can use strategies, but younger children cannot develop them independently (Pérez and Capilla, 2008; Rossignoli-Palomeque et al., 2020), although we can train them (Rossignoli-Palomeque et al., 2019).

For memory strategies (remember, Figures 7A,B), we only found differences for the low working memory load condition. The youngest children (KG and 2nd) reported using (or not) strategies in a similar proportion. However, the older children and college students’ proportion using memory strategies was higher than those who did not use any strategy. Fewer free cognitive resources to use strategies in the high-load conditions could explain the lack of modulations found for this more demanding condition.

However, we must consider that the way we studied strategies in the present work could also be related to the awareness of using them rather than using those strategies themselves. Maybe younger children are using these or other types of strategies to perform a task, but they simply are not aware of it or do not know how to explain their use verbally. This is consistent with the previous idea about the development of metacognitive knowledge dependent on language and other reasoning capacities under maturation during the early ages we tested here (Flavell, 1979; Efklides, 2008).

Notably, the present study’s findings have critical implications in educational contexts. One of the most relevant results is that even young children can perform a pretty inefficient object visual search task under high loads of working memory. However, we should adapt the time needed to complete the task successfully, and it depends on their stage of development in light of the present results. This is especially relevant in the case of the first years of formal education (elementary school) when time adaptations are essential for achievement.

Furthermore, regardless of age, all children will be more likely to commit more errors under high working memory loads, especially coming from distractors in a visual search. Therefore, we can try to reduce both working memory load and distractions/distractors by adaptations in activities in the classroom. Some strategies that a teacher can use in the classroom would be to let students write information to remember on paper. They may also choose to make information irrelevant to visual search but essential to other concurrent tasks available using pictograms and pictures or in writing. This way, students can refer to it when they need it without overloading their working memory. Moreover, these findings can be the basis for designing strategy training programs that will help children to perform search more efficiently, especially in the case of younger children who are not capable of self-generating themselves.

These recommendations are particularly important in children with executive function impairments, i.e., in children with language (Im-Bolter et al., 2006; Pascual-Leone and Johnson, 2021) or reading (Koltermann et al., 2020) impairments, arithmetic learning disabilities (Abreu-Mendoza et al., 2018), or attention deficit disorder with hyperactivity (Koltermann et al., 2020; Soto et al., 2021). The Theory of Constructive Operators and, specifically, the Model of Endogenous Mental Attention (Pascual-Leone and Johnson, 2005, 2021) have proven to be valid models to explain higher cognitive functioning and its development, as we have seen here, and for visual search. It would be helpful to explore their applications in tasks like those we used here on samples with atypical development or executive function problems (e.g., ADHD, dyslexia, specific language impairment, or learning disabilities).

Although this first study sheds some light on the role of working memory in visual search throughout childhood under a dual-task paradigm, additional research is needed to explore other relationships between the two processes. As we mentioned before, we did not manipulate any relationship between working memory contents and distractors/target in the visual search task like other studies have done in adulthood (e.g., Downing and Dodds, 2004; Gil-Gómez de Liaño et al., 2011). Allowing for the overlapping of stimuli between both tasks would be an interesting manipulation to test during childhood as both working memory and attention are under development between 5 and 12 years old (e.g., Brooking et al., 2012; Gil-Gómez de Liaño et al., 2020). It would also be interesting to study how search strategies like those used by Smilek et al. (2006) would apply to a developing brain.

## Conclusion

In summary, our study provides an explanation of visual working memory load modulation in the visual search task based on the neurodevelopmental perspective of the Model of Endogenous Mental Attention, replicating previous results in adulthood with some new effects. Moreover, our findings contribute to a better understanding of the use of metacognitive strategies during the development of visual search and the role of the individual differences that could mediate performance. The results provide some hints to consider in the educational context.

## Data availability statement

The raw data supporting the conclusions of this article will be made available by the authors, without undue reservation, to any qualified researcher.

## Ethics statement

The study was approved by the Ethics Committee of Universidad Autónoma de Madrid (CEI-84-1553). Written informed consent to participate in this study was provided by the participants’ legal guardian/next of kin.

## Author contributions

MQ-G, BG-GL, and EP-H conceived and planned the design, performed the data analysis, and wrote the manuscript. MQ-G programmed the experiments and carried out the data collection. All authors contributed to the article and approved the submitted version.

## Conflict of interest

The authors declare that the research was conducted in the absence of any commercial or financial relationships that could be construed as a potential conflict of interest.

## Publisher’s note

All claims expressed in this article are solely those of the authors and do not necessarily represent those of their affiliated organizations, or those of the publisher, the editors and the reviewers. Any product that may be evaluated in this article, or claim that may be made by its manufacturer, is not guaranteed or endorsed by the publisher.
