# Supplementary material for: I can look for it! Modulation of a concurrent Visual Working Memory task in Visual Search in development
Source: Front Psychol. 2022 Jul 22;13:907121. doi: 10.3389/fpsyg.2022.907121 (PMC9353270; doi:10.3389/fpsyg.2022.907121)
Supplement: Supplementary file 1 [file Data_Sheet_1.PDF]

frontiers

Supplementary Material 1. Working Memory task analysis

1 Data analysis

Data analysis of correct responses in the Working Memory (WM) task was conducted using generalized linear mixed-effects models (GLMM), similar to the one used to analyze the correct responses in the Visual Search task. In level 1, the independent variable was the WM load (Low and High). Grade was a predictor in level 2.

2 Results

Mean proportion of correct responses in the WM task is presented in Supplementary Figure 1 for each group of age and for the two WM load conditions.

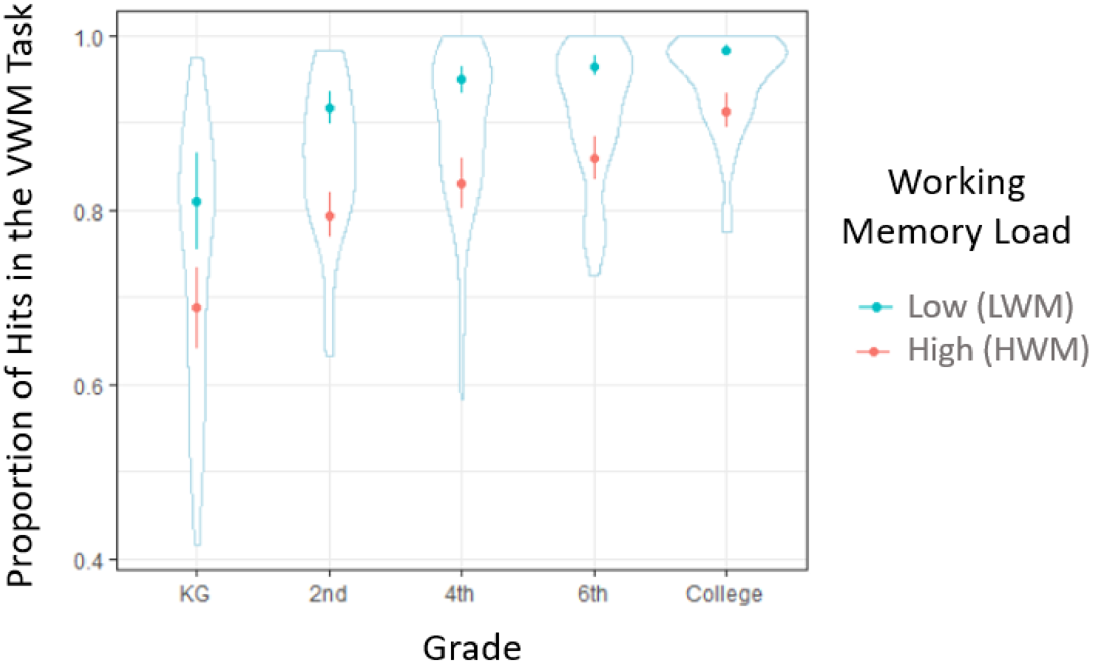

Supplementary Figure 1. Mean proportion of correct responses in the WM Task as a function of Grade and WM condition. Bars represent the confidence intervals for each mean value represented by the dots.

As we can see, as age/grade increases, the proportion of correct responses increases. Also, the proportion of correct responses was higher for the Low load condition. Indeed, the analysis showed significant main effects for both variables, WM and Grade, including the interaction (Supplementary Table 1). The interaction is essentially showing that changes between grades are more dramatic between Kindergarten and 2nd graders in both WM conditions, especially under Low WM (see confidence intervals in Supplementary Figure 2) showing a more logistic function with fewer

changes among older ages. For the High WM condition, the function shows still a bigger change between 6th children and young adults of the sample.

Supplementary Table 2. Estimated coefficients for Correct Responses (Odds Ratios) in the Working Memory task.

Fixed effects - Predictors	**Odds Ratios**	**SE**	**t**	**p**
Intercept (KG, WM Low)	5.37	0.67	13.46	**<0.001**
Working Memory (High)	0.47	0.03	-11.50	**<0.001**
Grade (2nd)	2.69	0.48	5.53	**<0.001**
Grade (4th)	4.91	0.89	8.74	**<0.001**
Grade (6th)	6.81	1.28	10.18	**<0.001**
Grade (College)	16.98	3.66	13.14	**<0.001**
Working Memory (High) x Grade (2nd)	0.67	0.07	-3.77	**<0.001**
Working Memory (High) x Grade (4th)	0.47	0.05	-6.55	**<0.001**
Working Memory (High) x Grade (6th)	0.42	0.05	-6.81	**<0.001**
Working Memory (High) x Grade (College)	0.31	0.05	-7.07	**<0.001**
Random Effects				
σ^2	3.29			
$\tau_{00\ Subject}$	0.32			
Intraclass Correlation Coefficient	0.09			
Marginal R^2 / Conditional R^2	0.231 / 0.299			

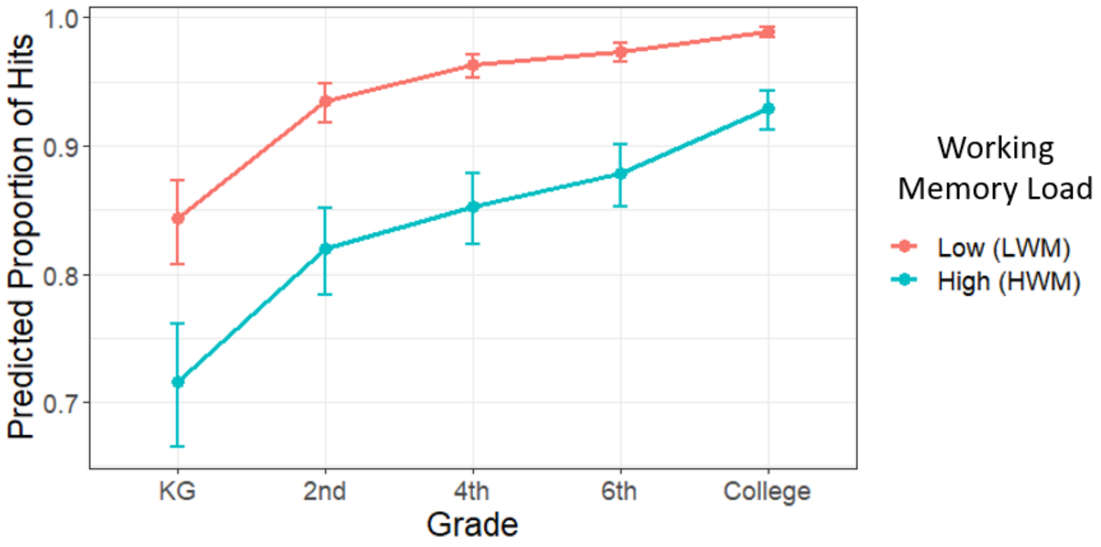

Supplementary Figure 2. Estimated Proportion of Correct Responses in the Working Memory task as a function of Grade and Target. Bars represent confidence intervals for each estimation (dots).
