# Supplementary material for: I can look for it! Modulation of a concurrent Visual Working Memory task in Visual Search in development
Source: Front Psychol. 2022 Jul 22;13:907121. doi: 10.3389/fpsyg.2022.907121 (PMC9353270; doi:10.3389/fpsyg.2022.907121)
Supplement: Supplementary file 2 [file Data_Sheet_2.PDF]

Supplementary Material 2. Building Progression of the Final Models

1 Building Progression Tables

Note. AIC = Akaike's information criterion; BIC = Schwarz's Bayesian Criterion; df = degrees of freedom; ICC = Intraclass Correlation Coefficient.

Supplementary Table 1. Building Progression of the Final Model for Accuracy – Correct Responses.

Step	Correct Responses	AIC	BIC	$\chi^2_{-2LL\ Change}$	df	p
	Null Model (ICC=.17)	14856	14873			
1	+ Target, Set size, Working Memory, Grade	12608	12682	2262.59	7	**<.0001**
2 (Final Model)	+ Target x Set size, Target x Grade, Set Size x Grade	12525	12674	100.59	9	**<.0001**
	Maximal Model	12540	12880	31.35	23	.12

Supplementary Table 2. Building Progression of the Final Model for False Alarms.

Step	False Alarms	AIC	BIC	$\chi^2_{-2LL\ Change}$	df	p
	Null Model (ICC=.29)	1999.1	2014.3			
1 (Final Model)	+ Working Memory, Set Size, Grade	1969.7	2030.7	41.381	6	**<.001**
	Maximal Model	1977	2137	18.71	13	.13

Supplementary Table 3. Building Progression of the Final Model for Commissions.

Step	Commissions	AIC	BIC	$\chi^2_{-2LL\ Change}$	df	p
	Null Model (ICC=.49)	840.32	855.54			
1 (Final Model)	+ Working Memory, Grade	822.91	876.18	27.41	5	**<.0001**
	Maximal Model	839.16	998.97	11.75	14	.627

Supplementary Table 4. Building Progression of the Final Model for Omissions.

Step	Omissions	AIC	BIC	$\chi^2_{-2LL\ Change}$	df	p
	Null Model (ICC=.18)	10666	10681			
1	+ Set size, Grade	10236	10289	439.79	5	**<.0001**
2 (Final Model)	+ Set size x Grade	10230	10314	13.85	4	**.007**
	Maximal Model	10238	10398	12.48	10	.25

Supplementary Table 5. Building Progression of the Final Model for Latency - Reaction Times.

Step	Reaction Times	AIC	BIC	$\chi^2_{-2LL\ Change}$	df	p
	Null Model (ICC=.24)	19499	19514			
1	+ Target, Set size, Working Memory	18116	18167	1397.17	7	**<.0001**
2 (Final Model)	+Target x Set size, Target x Grade, Set size x Grade, Working Memory x Grade	17760	17877	382.17	13	**<.0001**
	Maximal Model	17792	18005	5.73	19	.998

Supplementary Table 6. Building Progression of the Final Model for Search Slopes.

Step	Search Slopes	AIC	BIC	$\chi^2_{-2LL\ Change}$	df	p
	Null Model (ICC=.05)	5498.6	5511.7			
1 (Final Model)	+ Target	5016.4	5033.9	484.16	1	**<.0001**
	Maximal Model	5035.1	5131.4	17.36	18	.5

Supplementary Table 7. Building Progression of the Final Model for Efficiency Score.

Step	Efficiency Score	AIC	BIC	$\chi^2_{-2LL\ Change}$	df	p
	Null Model (ICC=.017)	-3898.3	-3883.1			
1	+ Target, Set size, Grade	-4622.4	-4576.8	736.107	6	**<.0001**
2 (Final Model)	+Target x Set size, Target x Grade	-4798.7	-4727.8	186.345	5	**<.0001**
	Maximal model	-4768.2	-4555.3	25.515	28	0.6
